# Repeatability of semi-quantitative [^68^Ga]Ga-FAPI-46 PET/CT measurements in pancreatobiliary cancers: a test-retest study

**DOI:** 10.1007/s00259-025-07738-6

**Published:** 2026-01-16

**Authors:** Rutger B. Henrar, Matthijs C. F. Cysouw, Xavier Palard-Novello, Lothar A. Schwarte, Pieter G. H. M. Raijmakers, Lioe-Fee de Geus-Oei, Alexander L. Vahrmeijer, Geert Kazemier, Albert D. Windhorst, Ronald Boellaard, Maqsood Yaqub, Daniela E. Oprea-Lager, Rutger-Jan Swijnenburg

**Affiliations:** 1 Amsterdam UMC location Vrije Universiteit Amsterdam, De Boelelaan 1117, Amsterdam, The Netherlands; 2 Cancer Center Amsterdam, Imaging and Biomarkers, Amsterdam, The Netherlands; 3 Univ Rennes, CLCC Eugène Marquis, INSERM, LTSI - UMR 1099, Rennes, France; 4 Leiden University Medical Center, Albinusdreef 2, Leiden, the Netherlands; 5 University of Twente, Drienerlolaan 5, Enschede, The Netherlands; 6 Delft University of Technology, Mekelweg 6, Delft, The Netherlands; 7 Radboud University Medical Center, Nijmegen, the Netherlands; 8 Department of Surgery, Amsterdam UMC location Vrije Universiteit Amsterdam, De Boelelaan 1117, Amsterdam, 1081 HV The Netherlands

**Keywords:** [^68^Ga]Ga-FAPI-46, LAFOV PET/CT, Test-retest, Repeatability, Pancreatobiliary cancer

## Abstract

**Purpose:**

Radiolabelled fibroblast activation protein inhibitors (FAPI) are promising molecular imaging tracers for the diagnosis and staging of pancreatobiliary cancer. To enable treatment response assessment with [^68^Ga]Ga-FAPI-46, the day-to-day variability of its uptake must be defined. This study aimed to determine the repeatability of semi-quantitative [^68^Ga]Ga-FAPI-46 PET/CT measurements in pancreatobiliary cancers.

**Methods:**

Patients with pathologically confirmed pancreatobiliary cancer (pancreatic ductal adenocarcinoma (PDAC) or cholangiocarcinoma (CCA)) were included. Patients underwent two [^68^Ga]Ga-FAPI-46 PET/CT scans, within 7 days, without any treatment before or between scans. Static long axial field-of-view (LAFOV) PET/CT scans were performed 60 min post-injection of [^68^Ga]Ga-FAPI-46 (EARL2-compliant reconstruction). Suspected malignant lesions were semi-automatically delineated (local background-adjusted 50% isocontour of the peak standardised uptake value (SUV)) and semi-quantitative measurements were extracted (SUV_mean_, SUV_peak_ and SUV_max_). Tumour-to-background ratios (TBR) were also calculated. Repeatability was assessed using the repeatability coefficient (RC) and the intraclass correlation coefficient (ICC).

**Results:**

Twelve patients were included (seven PDAC, three perihilar CCA and two intrahepatic CCA). The median injected dose was 216 MBq (interquartile range (IQR) 167–266 MBq) and the median uptake time was 60 min (range 60–68). In total, 70 FAPI-positive lesions were delineated. The RCs of SUV_mean_, SUV_peak_ and SUV_max_ were 23.7%, 23.9% and 29.8%, respectively. For the blood pool adjusted TBR_mean_, TBR_peak_ and TBR_max_, the RC’s were 21.6%, 20.8% and 28.0%, respectively. All ICCs were above 0.98.

**Conclusion:**

Semi-quantitative measurements of [^68^Ga]Ga-FAPI-46 PET/CT have an excellent repeatability and can potentially be used in future studies to assess treatment response in pancreatobiliary cancers.

**Supplementary Information:**

The online version contains supplementary material available at 10.1007/s00259-025-07738-6.

## Introduction

Radiolabelled fibroblast activation protein inhibitor (FAPI) tracers are a novel group of radiopharmaceuticals targeting fibroblast activation protein (FAP) on the cell surface of activated fibroblasts. In oncology, these fibroblasts are present in the tumour microenvironment and are referred to as cancer associated fibroblasts (CAFs). Multiple FAPI radiotracers have been developed for positron emission tomography (PET), with slightly different characteristics [1, 2]. Loktev et al. developed the [^68^Ga]Ga-FAPI-46 tracer, which showed high specificity, tumour retention and a fast predominantly renal clearance, resulting in high tumour-to-background ratios (TBR) [2]. In initial clinical trials, this tracer has shown promise to improve the diagnosis and staging in pancreatobiliary cancers, due to the high stromal content and presence of CAFs in these tumours [3–5].

Pancreatobiliary cancers, i.e. pancreatic ductal adenocarcinoma (PDAC) and cholangiocarcinoma (CCA), have a poor 5-year overall survival rate of approximately 9–13% [6]. Surgical resection is the only potentially curative treatment option. However, at diagnosis less than 20% of patients are eligible for surgery due to metastatic disease or the extent of vascular involvement [7]. Therefore, accurate staging and assessing treatment response are crucial to allocate the best suited treatment to patients and to improve outcomes [8–10]. Considering the expanding use of neoadjuvant therapy in pancreatobiliary cancers, the clinical need of accurately assessing treatment response has significantly increased [11–15].

Hybrid PET/computed tomography (CT) could provide the necessary cell-specific information in combination with anatomical information to improve the assessment of treatment response. Recent publications have shown the potential of 2-deoxy-2-[18F]fluoro-D-glucose ([^18^F]FDG) and FAPI uptake to assess treatment response after neoadjuvant therapy [16–19]. However, PET imaging with [^18^F]FDG has several downsides in comparison to FAPI tracers in pancreatobiliary tumours [5].

Our previous work validated the use of simplified quantification methods against full pharmacokinetic modelling for [^68^Ga]Ga-FAPI-46 in pancreatobiliary cancers [20]. To enable reliable treatment response assessment with [^68^Ga]Ga-FAPI-46, the day-to-day variability of [^68^Ga]Ga-FAPI-46 uptake must be determined. Therefore, this test-retest study aimed to determine the repeatability of semi-quantitative [68Ga]Ga-FAPI-46 PET/CT measurements in patients with pancreatobiliary cancer.

## Methods

This prospective test-retest study (PANSCAN-1, EudraCT Number 2022-001867-29) included twelve patients with histopathologically confirmed resectable, locally advanced or metastasized pancreatobiliary cancer, PDAC or CCA, between July 2024 and May 2025. Patients were eligible for inclusion if they were 18 years or older and if the primary tumour measured at least 20 mm on CT. Exclusion criteria were: inability to undergo a PET/CT scan, pregnancy or lactation, or impaired renal function (< 60 eGFR). All included patients signed an informed consent form after receiving verbal and written information on the study, according to local and (inter)national laws and regulations. The Medical Ethics Review Committee of the Amsterdam UMC reviewed and approved the study protocol (Institutional review board number 2022.0640).

### Data acquisition

Patients underwent two [^68^Ga]Ga-FAPI-46 PET/CT scans (on separate days) within 7 days and received no treatment prior to, nor in between, the test-retest scans. Patients were advised to drink 500–1000 ml of water before the scan. No fasting was required. The synthesis and quality control of [^68^Ga]Ga-FAPI-46 was performed as described by Palard et al. [20].

All [^68^Ga]Ga-FAPI-46 PET/CT scans were acquired on the same Biograph Vision Quadra system (Siemens Healthineers), a long axial field-of-view (LAFOV) PET/CT scanner, at the Amsterdam UMC, location VUmc (The Netherlands). The scan protocol and standardised uptake value (SUV) definition (body weight adjusted) were determined based on previous work using full pharmacokinetic modelling [20]. In short, a non-contrast enhanced low-dose CT scan (100 kV, with CARE Dose4D set to quality reference 7 mAs) was performed for attenuation correction and anatomical correlation, directly followed by a single bed position static whole-body (crown to mid-thigh) PET acquisition of 10 min, starting 60 min post-injection (p.i). The injected dose range was 100–345 megabecquerel (MBq), with a target dose of 300 MBq.

### Reconstruction protocol

The European Association of Nuclear Medicine Research Ltd. (EARL) 2-accredited reconstruction settings were used. An ordered subset expectation maximisation algorithm that incorporated time-of-flight and point-spread function modelling was used with the following parameters: 4 iterations, 5 subsets, decay correction, relative scaling scatter correction, CT-based attenuation correction, 4-mm Gaussian post-filtering, maximum full-ring difference of 85 crystal rings, and 3.30 × 3.30 × 2 mm^3^ voxel size.

### Data analysis

Each scan was independently interpreted by two physicians (nuclear medicine physician (PR) and senior nuclear medicine resident (MC)), who evaluated image quality and identified suspected malignant lesions. These lesions were semi-automatically delineated to obtain volumes of interest (VOI’s) using the in-house developed ACCURATE software based on a local background adjusted 50% isocontour of the peak standardised uptake value (SUVpeak, the highest mean SUV in a 12 mm diameter sphere in a VOI) [21]. Three background tissues were delineated: the blood pool (4 × 4 voxels were placed in five continuous slides in the ascending aorta), liver tissue (a 3 cm sphere was placed in non-diseased liver tissue, preferably the right liver lobe) and muscle tissue (a 2 cm sphere was placed in the m. psoas major) [22–24]. If necessary for the delineation of lesion, a mask was placed and mask-restricted region growing was applied to delineate a VOI. The standard-of-care triple phase contrast-enhanced CT scans were used as the reference imaging modality and were, if needed, used to assist the lesion delineation and mask placement.

The following PET parameters were collected from each individual VOI: the *volume* (cm^3^), the *standardised uptake values* (SUV, radioactivity concentration in a VOI corrected for the administered dose and body weight), *tumour-to-background ratios* (TBR, SUV metric divided by the SUV_mean_ of a background region) and *total lesion uptake* (TLU, SUV_mean_ or TBR_mean_ multiplied by the volume). Three SUV metrics were extracted: the SUV_mean_ (mean SUV in a VOI), the SUV_peak_ and the SUV_max_ (the highest SUV value of a single voxel in a VOI). For each background region (blood pool, liver and muscle tissue), a TBR was calculated for the three SUV metrics resulting in TBR_mean_, TBR_peak_ and TBR_max_.

For each patient, the *total tumour volume* (TTV, the sum of all delineated volumes per patient) and *total tumour burden* (TTB, the sum of all TLU’s per patient) were calculated.

Subgroup analyses of repeatability were performed for primary tumour lesions versus metastases and confirmed versus non-confirmed malignant lesions (i.e. lesions with histopathological proof of malignancy, the primary tumour lesion of a histopathologically confirmed metastasis or confirmed malignant lesion on an additional imaging modality). Furthermore, a comparison was made based on the median of the mean lesion volume of suspected metastases.

### Statistical analysis

Categorical variables were presented as frequencies and percentages. Continuous variables were presented as a mean with standard deviation (SD), if normally distributed. Otherwise, they were presented as a median with interquartile range (IQR). To assess the repeatability of the semi-quantitative measurements, the following metrics were calculated:

$$\:\mathrm{R}\mathrm{e}\mathrm{l}\mathrm{a}\mathrm{t}\mathrm{i}\mathrm{v}\mathrm{e}\:\mathrm{t}\mathrm{e}\mathrm{s}\mathrm{t}-\mathrm{r}\mathrm{e}\mathrm{t}\mathrm{e}\mathrm{s}\mathrm{t}\:\mathrm{d}\mathrm{i}\mathrm{f}\mathrm{f}\mathrm{e}\mathrm{r}\mathrm{e}\mathrm{n}\mathrm{c}\mathrm{e}\:\left(\mathrm{R}\mathrm{T}\mathrm{D}\right):\:(\mathrm{s}\mathrm{c}\mathrm{a}\mathrm{n}\:2-\:\mathrm{s}\mathrm{c}\mathrm{a}\mathrm{n}\:1)/\left(\right(\mathrm{s}\mathrm{c}\mathrm{a}\mathrm{n}\:1+\:\mathrm{s}\mathrm{c}\mathrm{a}\mathrm{n}\:2)/2)\:\mathrm{*}100$$

$$\:\mathrm{R}\mathrm{e}\mathrm{p}\mathrm{e}\mathrm{a}\mathrm{t}\mathrm{a}\mathrm{b}\mathrm{i}\mathrm{l}\mathrm{i}\mathrm{t}\mathrm{y}\:\mathrm{c}\mathrm{o}\mathrm{e}\mathrm{f}\mathrm{f}\mathrm{i}\mathrm{c}\mathrm{i}\mathrm{e}\mathrm{n}\mathrm{t}\:\left(\mathrm{R}\mathrm{C}\right):\:1.96\:\mathrm{*}\:\mathrm{S}\mathrm{D}\:\mathrm{o}\mathrm{f}\:\mathrm{R}\mathrm{T}\mathrm{D}$$

Bland-Altman plots were used to graphically present the RTDs. The single measure intraclass correlation coefficients (ICC) with 95% confidence intervals (two-way mixed model with an absolute agreement) were also calculated to assess repeatability of the test-retest measurements.

The Pitman-Morgan test was applied to assess differences in variance between paired test-retest measurements (correlated samples) [25]. The Holms-Bonferroni method was used to correct for multiple comparisons [26]. The Levene test was used in the subgroup analyses to compare variances between independent groups [27]. Statistical significance was defined as a p-value less than 0.05. SPSS (version 28.0.1.1) and R-studio (version 2024.12.1) were used for the statistical analyses. GraphPad Prism (version 10.2.0) was used for the graphical presentation of the data.

## Results

### Cohort description

Twelve patients were included: seven patients with PDAC, three patients with perihilar CCA and two patients with intrahepatic CCA. Table 1 presents the patient and scan characteristics of the included patients.

**Table 1:** Patient and scan characteristics of included patients (*n* = 12)

Characteristic	Data
Age	65 (59–74)
Sex (Male)	9 (75%)
Length (cm)	178 (170–185)
Weight (kg)	77 (65–90)
Disease stage	
Resectable	6 (50%)
Locally advanced	1 (8%)
Metastasized	5 (42%)
Injected activity (MBq)	
Test	214 (165–241)
Retest	231 (178–280)
Test-retest	216 (167–266)
Uptake time (min)	
Test (range)	60 (60–68)
Retest (range)	60 (60–68)
Test-retest differences	
Injected activity (MBq)	24 (− 44–62)
Interval test-retest (days)	3 (1–7)
Uptake time (min)	0 (0–0)

Categorial variables are presented as numbers with percentage in parentheses. Continuous variables are presented as medians with interquartile range in parenthesis, unless otherwise specified

The median injected activity was 216 MBq (IQR: 167–266). All patients, except two, underwent scanning at 60 min p.i. for both the test and retest scans. The two exceptions were scanned at 64 and 68 min p.i. for both scans. The median interval between the scans was three days (IQR: 1–7 days). One patient was scanned at an interval of 8 days. These deviations were due to logistical reasons. One patient was excluded from the analysis of the liver background uptake, due to unsolved biliary obstruction resulting in the retention of the tracer in the liver parenchyma (Supplemental Fig. 1B). All scans were performed on the same LAFOV PET/CT scanner. An example of test-retest images is shown in Supplemental Fig. 2A-B.

### Lesions

A total of 70 lesions were delineated: 12 primary tumour lesions, 56 metastases (20 lymph node, 20 liver, 14 peritoneal and two lung metastases) and two newly detected second primary tumours. The median number of lesions per patient was five (IQR: 1–7). Twenty lesions were confirmed malignant lesions: 16 histopathologically confirmed lesions, three primary tumour lesions of a histopathologically confirmed metastasis and one confirmed malignant lesion on an additionally performed imaging modality. One of the newly detected second primary tumours was confirmed with histopathology (distal oesophageal carcinoma), the other was confirmed with an additional imaging modality (breast carcinoma). In the combined test-retest scans, the median volume of all lesions was 3.5 cm^3^ (IQR: 2.0–7.3). The median volume of primary tumour lesions was 21.4 cm^3^ (IQR: 8.9–34.3) and was 2.9 cm^3^ (IQR: 1.9–4.6) for metastases. Table 2 shows the descriptive values of the analysed PET parameters of the test and retest scans.

**Table 2:** Descriptive data of the analysed PET parameters of the test and retest scans

	Test		Retest	
Parameter	Median	IQR	Median	IQR
Volume	3,3	1,9 − 7,4	3,5	2,0–7,3
TTV	33,7	18,5–47,6	36,5	20,0–51,2
SUV_mean_	4,0	2,8 − 6,7	4,0	3,0–6,7
SUV_peak_	4,8	2,9 − 8,3	4,8	3,3–8,6
SUV_max_	6,2	4,4–10,8	6,7	4,6–10,2
TLU SUV	14,7	4,4–33,7	15,1	6,5–32,9
TTB SUV	249,8	105,7–503,4	252,6	121,0–502,2
Blood pool				
Background	1,4	1,2 − 1,5	1,4	1,3 − 1,5
TBR_mean_	2,7	2,0–4,0	2,7	2,1–4,8
TBR_peak_	3,1	2,2–4,9	3,2	2,4–5,9
TBR_max_	4,3	3,1–6,6	4,5	3,3–7,2
TLU TBR	9,0	3,4–25,5	10,4	4,6–24,2
TTB TBR	174,8	84,3–363,8	172,8	85,5–368,2
Liver tissue *				
Background	1,9	1,3 − 2,2	1,9	1,5 − 2,0
TBR_mean_	2,3	1,6 − 3,4	2,1	1,7 − 3,6
TBR_peak_	2,7	1,8 − 4,2	2,7	2,0–4,5
TBR_max_	3,7	2,4–5,4	3,5	2,6 − 5,7
TLU TBR	9,1	3,7–23,4	8,4	5,0–23,4
TTB TBR	131,9	55,6–408,9	141,5	51,1–413,6
Muscle Tissue				
Background	0,8	0,7 − 0,9	0,8	0,8 − 1,0
TBR_mean_	5,0	3,7–8,9	5,0	3,3–8,6
TBR_peak_	6,1	4,0–10,9	6,2	3,5–10,7
TBR_max_	7,9	5,7–13,7	8,2	5,1–13,6
TLU TBR	18,6	6,3–41,2	19,2	6,8–41,7
TBB TBR	297,4	108,7–721,3	318,1	118,1–652,2

Abbreviations: IQR: interquartile range, TTV: total tumour volume, SUV: standardised uptake value, TLU: total lesion uptake, TTB: total tumour burden, TBR: tumour-to-background ratio

* One patient with unsolved biliary obstruction was excluded from the analysis

### Repeatability of semi-quantitative metrics

**Fig. 1:**
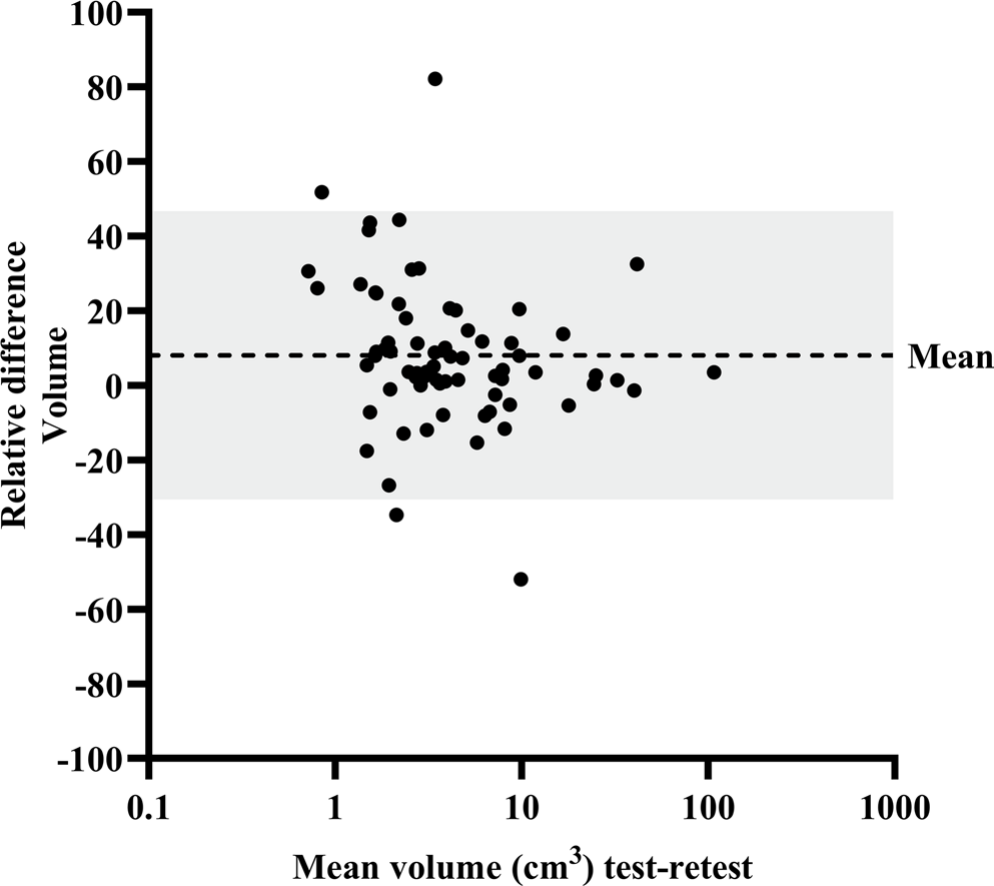
Bland-Altman plot of the relative test-retest differences in lesion volume measurements. Each dot represents one lesion (*n* = 70). The dashed line shows the mean difference (bias), while the shaded area indicates the 95% limits of agreement. The x-axis is log-scaled

**Fig. 2:**
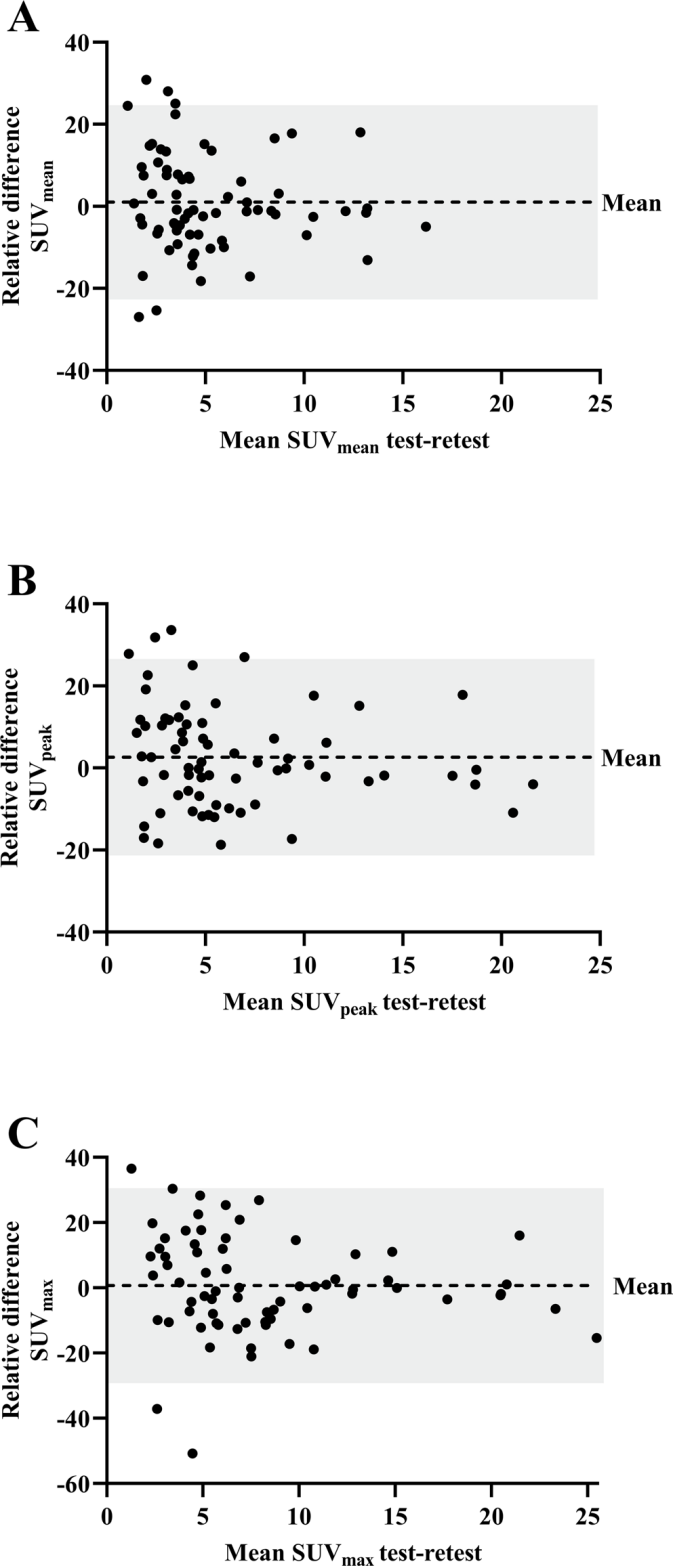
**A-C** Bland-Altman plots of the relative test-retest differences of the standardised uptake value (SUV) metrics: SUV_mean_ (A), SUV_peak_ (B) and SUV_max_ (C). Each dot represents one lesion (*n* = 70). The dashed line shows the mean difference (bias), while the shaded area indicates the 95% limits of agreement

**Fig. 3:**
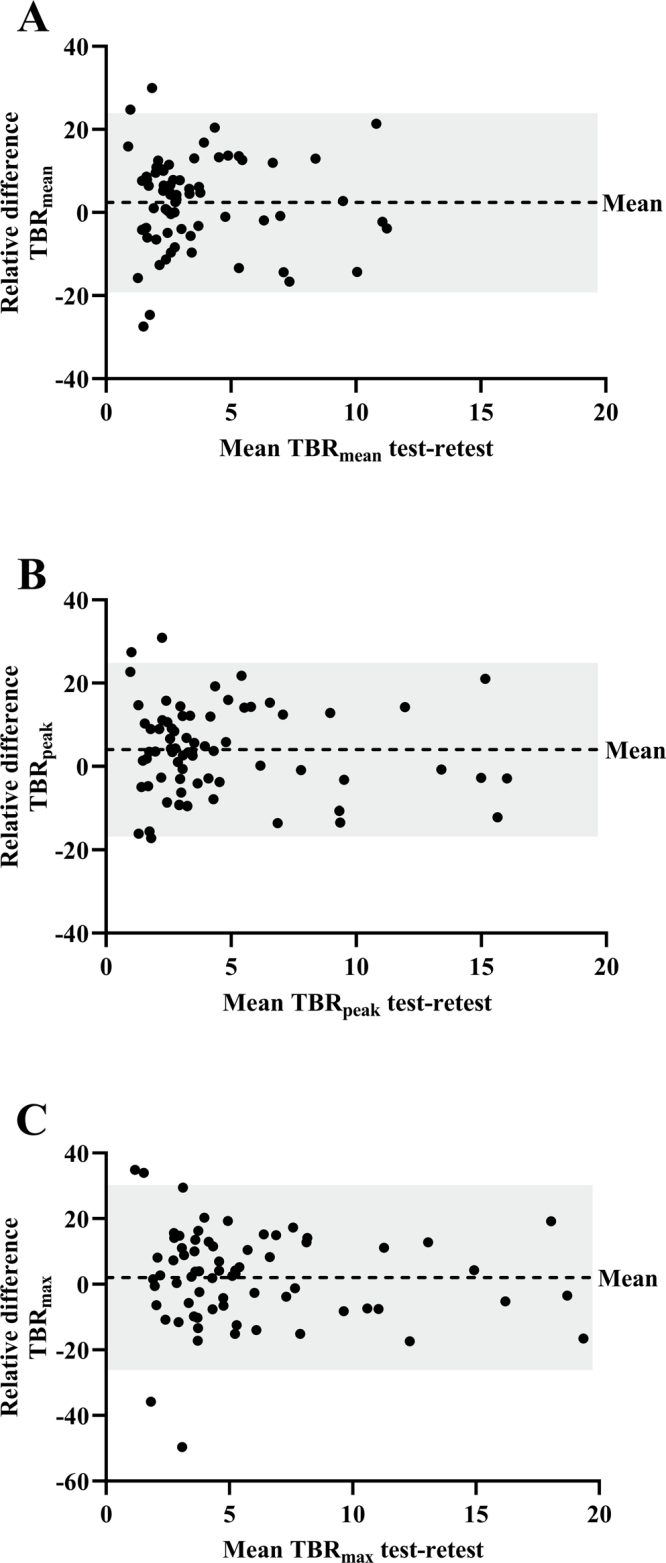
**A-C** Bland-Altman plots of the relative test-retest differences in blood pool adjusted tumour-to-background ratios (TBR): TBR_mean_ (A), TBR_peak_ (B) and TBR_max_ (C). Each dot represents one lesion (*n* = 70). The dashed line shows the mean difference (bias), while the shaded area indicates the 95% limits of agreement

The RC of the semi-automatic volume measurements was 38.6% (Fig. 1). The highest repeatability of the SUV metrics was observed for SUVmean (RC 23.7%), followed by SUVpeak (RC 23.9%) and SUVmax (RC 29.8%). The RTD of the SUV metrics are presented in Fig. 2A-C. Of the three background tissues, muscle tissue showed the highest repeatability, with a RC of 18.3%, followed by the blood pool (19.1%) and liver tissue (21.8%) (Supplemental Fig. 1A-C). The blood pool adjusted TBRs demonstrated the highest repeatability, in comparison to the muscle and liver tissue adjusted TBRs (Table 3). For the blood pool adjusted TBRs, the RTDs are presented in Fig. 3A-C and the RTDs against the mean lesion volume are presented in Supplemental Fig. 3A-C. The RC of TLU of the SUV and the blood pool adjusted TBR was 38.8% and 34.5%, respectively (Supplemental Fig. 4A-B). The variances of the blood pool adjusted TLU and volume measurements were statistically significantly different between the test-retest scan (*p* < 0.001). Table 4 shows an overview of the relative test-retest differences of the best performing PET parameters. All semi-quantitative measures showed excellent ICCs (Table 4). Overall, the blood pool adjusted TBR_mean_ (RC 21.6%) and TBR_peak_ (RC 20.8%) demonstrated the highest repeatability of all semi-quantitative measures.

**Table 3:** Comparison of the relative test-retest differences of the three background adjusted tumour-to-background ratios (TBRs)

Parameter	Mean	SD	RC	Lower Limit	Upper Limit	ICC (95% CI)
Blood pool						
Background	2,6	9,8	19,2	−16,5	21,7	0,69 (0,23 − 0,90)
TBR_mean_	2,4	11,0	21,6	−19,1	24,0	0,98 (0,97 − 0,99)
TBR_peak_	4,0	10,6	20,8	−16,8	24,8	0,98 (0,98 − 0,99)
TBR_max_	2,1	14,3	28,0	−26,1	30,2	0,98 (0,97 − 0,99)
TLU TBR	10,5	17,6	34,5	−23,9	44,9	0,99 (0,98 − 0,99)
TTB TBR	2,9	11,6	22,7	−19,9	25,6	0,98 (0,94 − 1,00)
Liver tissue*						
Background	0,3	11,1	21,8	−21,5	22,0	0,93 (0,75 − 0,98)
TBR_mean_	2,0	19,7	38,6	−36,0	40,5	0,98 (0,96 − 0,99)
TBR_peak_	3,4	19,2	37,6	−34,1	41,0	0,98 (0,97 − 0,99)
TBR_max_	1,3	22,1	43,3	−42,1	44,7	0,97 (0,95 − 0,98)
TLU TBR	8,8	24,0	47,0	−38,3	55,8	1,00 (1,00–1,00)
TTB TBR	3,2	17,6	34,5	−31,3	37,7	1,00 (0,99 − 1,00)
Muscle tissue						
Background	6,4	9,3	18,2	−11,9	24,7	0,77 (0,30 − 0,93)
TBR_mean_	−4,5	13,6	26,7	−31,2	22,2	0,97 (0,95 − 0,98)
TBR_peak_	−2,8	13,5	26,5	−29,2	23,6	0,97 (0,95 − 0,98)
TBR_max_	−4,8	16,8	32,9	−37,7	28,2	0,96 (0,93 − 0,97)
TLU TBR	3,6	18,4	36,1	−32,2	39,6	1,00 (1,00–1,00)
TBB TBR	0,9	13,0	25,5	−26,4	24,5	1,00 (0,99 − 1,00)

Abbreviations: SD: standard deviation, RC: repeatability coefficient, Lower and Upper limit: lower and upper limits of agreement, ICC: intraclass correlation coefficient (single measure, two way mixed model with absolute agreement), CI: confidence interval, TBR: tumour-to-background ratio, TLU: total lesion uptake, TTB: total tumour burden

* One patient with unsolved biliary obstruction was excluded from the analysis

**Table 4:** Overview of the relative test-retest differences of SUV metrics and blood pool adjusted TBRs for the lesion and patient based PET parameters

Parameter	Mean	SD	RC	Lower Limit	Upper Limit	ICC (95% CI)
Lesion based						
Volume	8,1	19,7	38,6	−30,5	46,7	0,99 (0,99 − 1,00)
SUV_mean_	1,0	12,1	23,7	−22,6	24,6	0,98 (0,98 − 0,99)
SUV_peak_	2,7	12,2	23,9	−21,3	26,6	0,99 (0,98 − 0,99)
SUV_max_	0,7	15,2	29,8	−29,1	30,5	0,98 (0,97 − 0,99)
TBR_mean_	2,4	11,0	21,6	−19,1	24,0	0,98 (0,97 − 0,99)
TBR_peak_	4,0	10,6	20,8	−16,8	24,8	0,98 (0,98 − 0,99)
TBR_max_	2,1	14,3	28,0	−26,1	30,2	0,98 (0,97 − 0,99)
TLU SUV	9,1	19,8	38,8	−29,7	47,9	1,00 (1,00 – 1,00)
TLU TBR	10,5	17,6	34,5	−23,9	44,9	0,99 (0,98 − 0,99)
Patient based						
TTV	4,6	10,4	20,4	−15,9	25,0	1,00 (0,98 − 1,00)
TTB SUV	5,5	13,0	25,5	−20,0	31,0	1,00 (0,99 − 1,00)
TTB TBR	2,9	11,6	22,7	−19,9	25,6	0,98 (0,94 − 1,00)

Abbreviations: SD: standard deviation, RC: repeatability coefficient, Lower and Upper limit: lower and upper limits of agreement, ICC: intraclass correlation coefficient (single measure, two way mixed model with absolute agreement), CI: confidence interval, SUV: standardised uptake value, TBR: tumour-to-background ratio (blood pool adjusted), TLU: total lesion uptake, TTB: total tumour burden, TTV: total tumour volume

### Patient based parameters

The highest repeatability of the TTB was observed for the blood pool adjusted TBR (RC 22.7%), followed by the SUV and muscle tissue adjusted TBR (RC both 25.5%), and the liver tissue adjusted TBR (RC 33.9%). The RC of the sum of all volumes (TTV) was 20.4% (Supplemental Fig. 5A-C).

### Subgroup analyses

First, the repeatability of the semi-quantitative measures was compared between primary tumour lesions and metastases. The largest difference in RCs was observed for the volume measurements (23.5% vs. 41.6% for primary lesions vs. metastases, respectively). The median absolute difference in volumes was 0.6 cm^3^ (IQR: −0.4–2.2) and 0.2 cm^3^ (IQR: 0.0–0.4), respectively. The repeatability of the SUV metrics and blood pool adjusted TBRs were comparable between the subgroups, the largest difference in RCs was observed for SUV_max_ (24.7% vs. 31.4%) and TBR_peak_ (24.1% vs. 20.0%) (Supplemental Table 1). Secondly, a comparison was performed between confirmed and non-confirmed malignant lesions. Similarly, the largest difference was observed in the volume measurements (25.3% vs. 42.9%). The differences for the SUV metrics and blood pool adjusted TBRs were also comparable. The largest difference in RC was observed for both SUV_max_ (26.5% vs. 31.0%) and TBR_peak_ (19.6% and 24.1%) (Supplemental Table 2). Lastly, a comparison was made based on the median lesion size of metastases: ≤ 2.9 cm^3^ and > 2.9 cm^3^. The differences in RC were small in most semi-quantitative measures (range − 2.2% − 3.7%). The SUV_max_ and the blood pool adjusted TBR_max_ were the exceptions, with a difference in RC of 11.0% (24.9% vs. 35.9%) and 9.6% (23.7% versus 33.3%), respectively (Supplemental Table 3). No statistically significant difference in variance was found for all subgroups.

## Discussion

This study investigated the day-to-day variability of [^68^Ga]Ga-FAPI-46 uptake and volumetric measurements in a test-retest setting, to determine what percentage change in [^68^Ga]Ga-FAPI-46 uptake most likely constitutes measurement noise and what percentage change most likely constitutes a true biological effect. This is an essential step towards the application of FAPI PET imaging for assessing treatment response.

Our results show that the semi-quantitative measurements of [^68^Ga]Ga-FAPI-46 uptake have an excellent repeatability. Overall, the blood pool adjusted TBRs demonstrated better repeatability than the SUV metrics. This is in accordance with our previous work, which showed that the blood pool adjusted TBR_mean_ had the strongest correlation with the full pharmacokinetic model (r_s_: 0.877) [20]. Since TBR has a better repeatability than SUV, image-derived blood activity concentrations (used for TBR calculations) may (at least partly) correct for variability in tumour tracer uptake that is not accounted for when using injected dose in SUV calculations. However, for the clinical application of [^68^Ga]Ga-FAPI-46 PET/CT as a response monitoring modality, a post-therapy kinetic analysis may be indicated for treatments that could significantly alter tracer kinetics to confirm the use of semi-quantitative measures. We showed that the blood pool adjusted TBR_peak_ is the best suited measure to assess uptake over time. Hence, any increase or decrease in TBR_peak_ greater than 21% likely indicates a significant change in FAP expression, which in turn may reflect disease progression or treatment response, respectively. The volume (derived) measurements were the worst performing semi-quantitative measures. This was primarily due to the relatively small volumes of metastatic lesions, where a small absolute difference resulted in a large relative effect on the repeatability. However, when all volume measurements per patient were combined (TTV), the repeatability was high (20.4%).

This is the first study to determine the day-to-day variability of a FAPI tracer. The [^68^Ga]Ga-FAPI-46 tracer was investigated, because of its favourable imaging characteristics in comparison to other commonly used FAPI tracers (FAPI-04 and FAPI-74) and the promising results in pancreatobiliary cancers [2–4]. A recent study investigated the reproducibility of semi-quantitative measurements with [^68^Ga]Ga-FAPI-04 multi-timepoint imaging (at 15 and 60 min p.i.) in various malignancies and showed high correlations for these measurements. Although, this is different concept than test-retest repeatability, these results indicate the potential of quantitative measurements with FAPI tracers [28]. In the literature, few studies have assessed the variability of the uptake of tumour-specific PET tracers. An example of such a study was conducted in prostate cancer patients. Jansen et al. investigated a prostate-specific membrane antigen (PSMA) PET tracer and reported RCs of 24.4%, 25.3% and 31.0% for SUVmean, SUV peak and SUVmax, respectively [22]. For the blood pool adjusted TBRs, they reported RCs ranging from 31.7% to 37.3%.

The most data on the variability of tracer uptake in PET imaging derives from [^18^F]FDG PET research. A meta-analysis found that the main factors influencing the repeatability were the location of a study (i.e. mono or multicenter study) and which semi-quantitative measure was used (SUV_max_, SUV_peak_ or SUV_mean_) [29]. They found the SUV_peak_ measurement to be the most precise and reproducible in [^18^F]FDG PET imaging, as this metric was less influenced by variations in image quality and segmentation uncertainties. Another review by Martin Lodge analysed the results of test-retest studies of [^18^F]FDG PET imaging in various oncological diseases [30]. Based on these results (the within-subject coefficients of variation), we calculated the RCs for [^18^F]FDG PET imaging. Overall, our results seem to be in line with the repeatability of [^18^F]FDG and PSMA PET imaging for SUVmax (29.8% vs. 30.4% and 31.0%), SUV_peak_ (23.9% vs. 27.7% and 25.3%) and SUV_mean_ (23.7% vs. 26.6% and 24.4%). In accordance with these PET radiotracers, we found that the uptake variability was higher in small and in low-uptake lesions, probably due to image noise, segmentation uncertainties, motion artifacts and potential differences in partial volume effects.

The limited ability of anatomical imaging modalities (contrast-enhanced CT and magnetic resonance imaging (MRI)) to assess treatment response after chemotherapy is mostly attributed to the difficulty to differentiate fibrosis from viable tumour tissue and the overestimation of vascular involvement [15, 31]. After neoadjuvant or induction chemotherapy, anatomical criteria in combination with biological and conditional criteria are used to guide clinical decision making regarding the optimal treatment for each patient: best supportive care, continuation or change of chemotherapy, or surgical exploration and resection. However, in patients with locally advanced PDAC, this assessment results in 28% of the surgical explorations being futile (non-therapeutic) [32]. These outcomes have driven efforts to develop imaging biomarkers that could improve the treatment response assessment and clinical decision making. Therefore, potentially improving the patient selection for surgery and patient outcomes by reducing futile explorations. An example of such an imaging biomarker study was conducted by Abdelrahman et al. [16]. They investigated the use of [^18^F]FDG PET for assessing treatment response in PDAC patients and found it was promising to predict pathological response. However, no quantification of the change in [^18^F]FDG uptake was performed and not all patients underwent a pre-therapy scan. Additionally, [^18^F]FDG has limitations in comparison to the FAPI tracer and its repeatability is influenced by more factors, such as diabetes mellitus and varying blood glucose levels [5, 33, 34]. Recent studies have shown that the uptake of FAPI decreases after treatment with chemo(radio)therapy [17–19]. Moreover, Li et al. found that the change in [^18^F]FAPI-04 uptake was associated with the grade of pathological treatment response [19]. Indicating the potential of FAPI tracers as an imaging biomarker to assess treatment response. However, without determining the variability of FAPI uptake, the interpretation of changes in uptake over time are severely flawed.

For [^18^F]FDG PET imaging, the PET Response Criteria in Solid Tumours (PERCIST) were developed to standardise the metabolic response monitoring [35]. The proposed criteria to define treatment response were based on the test-retest variability of [^18^F]FDG uptake. Partial metabolic response and progressive metabolic disease were defined as a change of at least 30% in SUL_peak_ (peak standardised uptake value adjusted for lean body mass) and an absolute change of at least 0.8 SUL_peak_. Our results could be used as a starting point for PERCIST-like criteria for FAPI PET imaging, using the repeatability coefficients of FAPI uptake to define likely biological changes in FAP expression and potential treatment effects. This would naturally require clinical validation in terms of pathological response and patient survival. We suggest using TBR_peak_ as it demonstrated the highest repeatability (20.8%) and our previous work on [^68^Ga]Ga-FAPI-46 pharmacokinetics indicated that TBR metrics were better suited for the quantification of [^68^Ga]Ga-FAPI-46 uptake than SUV metrics. Also, using the peak uptake seems optimal as it is known to be less sensitive to delineation method compared to the mean uptake and less sensitive to noise than the maximum uptake [36].

The main limitations of this study are the small sample size and its monocenter design. The applicability of our results to other centers and to other tumour types is yet unknown. Although, harmonised EARL2 reconstructions were used, the variability in other settings or with other (standard field-of-view) PET systems could be higher. We would therefore encourage others researchers to replicate our study at their institution(s) and in other tumour types to validate these findings. For the other commonly used FAPI tracers ([^68^Ga]Ga-FAPI-04 and [^18^F]-FAPI-74) no data on repeatability is currently available. While we may expect similar findings using these tracers in the same tumour types, the exact test-retest variability may differ and will need to be determined.

Regarding the different malignancies included in this study (PDAC, intrahepatic and perihilar CCA), we have previously confirmed the use of simplified quantitative measures of [^68^Ga]Ga-FAPI-46 uptake in these tumours and found no differences in variances between the tumour types in this study (analysis not reported).

Semi-quantitative PET measurements are inherently complicated by uptake variability due to technical, physical and biological variations [34]. Reducing, and ideally eliminating, any potential sources of variability is essential to ensure the reliability of test-retest results and of the assessment of uptake over time. Therefore, we ensured that the same scanner, imaging and reconstruction protocols were used. Future response assessment studies should also adhere to such protocols.

## Conclusion

This test-retest study assessed the repeatability of semi-quantitative measurements of [^68^Ga]Ga-FAPI-46 uptake in pancreatobiliary tumour lesions and showed excellent repeatability of all measures. The blood pool adjusted TBRs showed better repeatability in comparison to the SUV metrics, which aligns with the results of our previous pharmacokinetic validation study. The blood pool adjusted TBR_peak_ (20.8%) demonstrated the best RC. Thus, in pancreatobiliary cancers changes in TBR_peak_ exceeding 21% can be considered a likely biological change in FAP expression. While using TBR may partly correct for changes in plasma availability of the tracer, post-therapy kinetic analysis may be warranted for therapies that may significantly alter tracer kinetics. Our findings enable the clinical evaluation of [^68^Ga]Ga-FAPI-46 uptake as an imaging biomarker in pancreatobiliary cancer and may in the future improve the treatment response assessment after chemo(radio)therapy.

## Supplementary Information

Below is the link to the electronic supplementary material.

Supplementary Material 1

## Data Availability

The datasets generated during the current study are available from the corresponding author on reasonable request.

## Declarations

The precursor of FAPI-46 and reference standard [natGa]Ga-FAPI-46 (ABX, Radeberg, Germany) were made freely available.

## References

[1] Lindner T, Loktev A, Altmann A, Giesel F, Kratochwil C, Debus J, et al. Development of quinoline-based theranostic ligands for the targeting of fibroblast activation protein. J Nucl Med. 2018;59:1415–22. 10.2967/jnumed.118.210443. 29626119 10.2967/jnumed.118.210443

[2] Loktev A, Lindner T, Burger EM, Altmann A, Giesel F, Kratochwil C, et al. Development of fibroblast activation protein-targeted radiotracers with improved tumor retention. J Nucl Med. 2019;60:1421–9. 10.2967/jnumed.118.224469. 30850501 10.2967/jnumed.118.224469 PMC6785792

[3] Kessler L, Hirmas N, Pabst KM, Hamacher R, Ferdinandus J, Schaarschmidt BM, et al. (68)Ga-labeled fibroblast activation protein inhibitor ((68)Ga-FAPI) PET for pancreatic adenocarcinoma: data from the (68)Ga-FAPI PET observational trial. J Nucl Med. 2023;64:1910–7. 10.2967/jnumed.122.264827. 37973185 10.2967/jnumed.122.264827

[4] Pabst KM, Trajkovic-Arsic M, Cheung PFY, Ballke S, Steiger K, Bartel T, et al. Superior tumor detection for (68)Ga-FAPI-46 versus (18)F-FDG PET/CT and conventional CT in patients with cholangiocarcinoma. J Nucl Med. 2023;64:1049–55. 10.2967/jnumed.122.265215. 37024301 10.2967/jnumed.122.265215 PMC10315700

[5] Henrar RB, Vuijk FA, Burchell GL, van Dieren S, de Geus-Oei LF, Kazemier G, et al. Diagnostic performance of radiolabelled FAPI versus [(18)F]FDG PET imaging in hepato-pancreato-biliary oncology: a systematic review and meta-analysis. Int J Mol Sci. 2025. 10.3390/ijms26051978. 40076605 10.3390/ijms26051978 PMC11900289

[6] Institute NC. SEER*Explorer: an interactive website for SEER cancer statistics. National Cancer Institute; 2023.

[7] Huang L, Jansen L, Balavarca Y, Molina-Montes E, Babaei M, van der Geest L, et al. Resection of pancreatic cancer in Europe and USA: an international large-scale study highlighting large variations. Gut. 2019;68:130–9. 10.1136/gutjnl-2017-314828. 29158237 10.1136/gutjnl-2017-314828

[8] Cao J, Srinivas-Rao S, Mroueh N, Anand R, Kongboonvijit S, Sertic M, et al. Cholangiocarcinoma imaging: from diagnosis to response assessment. Abdom Radiol. 2024;49:1699–715. 10.1007/s00261-024-04267-y. 10.1007/s00261-024-04267-y 38578323

[9] Perrotta G, Mohamed G, Larson BK, Osipov A, Ferrone CR, Lo SK, et al. Accuracy of clinical staging in early-stage pancreatic ductal adenocarcinoma. JAMA. 2024;332:1108–10. 10.1001/jama.2024.16332. 39235802 10.1001/jama.2024.16332 PMC11378063

[10] Fiore M, Coppola A, Petrianni GM, Trecca P, D’Ercole G, Cimini P, et al. Advances in pre-treatment evaluation of pancreatic ductal adenocarcinoma: a narrative review. J Gastrointest Oncol. 2023;14:1114–30. 10.21037/jgo-22-1034. 37201095 10.21037/jgo-22-1034 PMC10186502

[11] Conroy T, Hammel P, Hebbar M, Ben Abdelghani M, Wei AC, Raoul JL, et al. FOLFIRINOX or gemcitabine as adjuvant therapy for pancreatic cancer. N Engl J Med. 2018;379:2395–406. 10.1056/NEJMoa1809775. 30575490 10.1056/NEJMoa1809775

[12] Goetze TO, Bechstein WO, Bankstahl US, Keck T, Konigsrainer A, Lang SA, et al. Neoadjuvant chemotherapy with gemcitabine plus cisplatin followed by radical liver resection versus immediate radical liver resection alone with or without adjuvant chemotherapy in incidentally detected gallbladder carcinoma after simple cholecystectomy or in front of radical resection of BTC (ICC/ECC) - a phase III study of the German registry of incidental gallbladder carcinoma platform (GR)- the AIO/ CALGP/ ACO- GAIN-trial. BMC Cancer. 2020;20:122. 10.1186/s12885-020-6610-4. 32059704 10.1186/s12885-020-6610-4 PMC7023745

[13] Ten Haaft B, Sickmann MMT, Nooijen LE, Ali M, Wilmink JW, Klumpen HJ, et al. Gemcitabine-cisplatin induction treatment in patients with locally advanced perihilar cholangiocarcinoma (IMPACCA): a prospective registration study. Eur J Surg Oncol. 2025;51:109358. 10.1016/j.ejso.2024.109358. 39638652 10.1016/j.ejso.2024.109358

[14] Versteijne E, van Dam JL, Suker M, Janssen QP, Groothuis K, Akkermans-Vogelaar JM, et al. Neoadjuvant chemoradiotherapy versus upfront surgery for resectable and borderline resectable pancreatic cancer: long-term results of the Dutch randomized PREOPANC trial. J Clin Oncol. 2022;40:1220–30. 10.1200/JCO.21.02233. 35084987 10.1200/JCO.21.02233

[15] Stoop TF, Theijse RT, Seelen LWF, Groot Koerkamp B, van Eijck CHJ, Wolfgang CL, et al. Preoperative chemotherapy, radiotherapy and surgical decision-making in patients with borderline resectable and locally advanced pancreatic cancer. Nat Rev Gastroenterol Hepatol. 2024;21:101–24. 10.1038/s41575-023-00856-2. 38036745 10.1038/s41575-023-00856-2

[16] Abdelrahman AM, Goenka AH, Alva-Ruiz R, Yonkus JA, Leiting JL, Graham RP, et al. FDG-PET predicts neoadjuvant therapy response and survival in borderline Resectable/Locally advanced pancreatic adenocarcinoma. J Natl Compr Canc Netw. 2022;20:1023–e323. 10.6004/jnccn.2022.7041. 36075389 10.6004/jnccn.2022.7041 PMC12001712

[17] Metzger G, Bayerl C, Rogasch JM, Furth C, Wetz C, Beck M. ^68^ Ga-labeled fibroblast activation protein inhibitor (FAPI) PET/CT for locally advanced or recurrent pancreatic cancer staging and restaging after chemoradiotherapy. Theranostics. 2024;14:4184–97. 10.7150/thno.95329. 39113796 10.7150/thno.95329 PMC11303068

[18] Zhu Z, Cheng K, Yun Z, Zhang X, Hu X, Liu J. [18F] AlF-NOTA-FAPI-04 PET/CT can predict treatment response and survival in patients receiving chemotherapy for inoperable pancreatic ductal adenocarcinoma. Eur J Nucl Med Mol Imaging. 2023;50:3425–38. 10.1007/s00259-023-06271-8. 37328622 10.1007/s00259-023-06271-8

[19] Li X, Lu N, Sun K, Shi F, Lin L, Chen Y, et al. [(18)F]FAPI- 04 PET/CT for pathologic response assessment in pancreatic cancer patients with systemic treatment. Eur J Nucl Med Mol Imaging. 2025. 10.1007/s00259-025-07271-6. 40237796 10.1007/s00259-025-07271-6

[20] Palard-Novello X, Henrar RB, Oprea-Lager DE, Cysouw MCF, Schober P, de Geus-Oei LF, et al. Assessment of fully quantitative and simplified methods for analysis of [(68)Ga]Ga-FAPI-46 uptake in patients with pancreatobiliary cancer using LAFOV PET/CT. Eur J Nucl Med Mol Imaging. 2025;52:1472–80. 10.1007/s00259-024-07037-6. 39743615 10.1007/s00259-024-07037-6

[21] Boellaard R. Quantitative oncology molecular analysis suite: ACCURATE [abstract]. J Nucl Med. 2018;59(suppl 1):1753.

[22] Jansen BHE, Cysouw MCF, Vis AN, van Moorselaar RJA, Voortman J, Bodar YJL, et al. Repeatability of quantitative (18)F-DCFPyL PET/CT measurements in metastatic prostate cancer. J Nucl Med. 2020;61:1320–5. 10.2967/jnumed.119.236075. 31924729 10.2967/jnumed.119.236075 PMC7456167

[23] Palard-Novello X, Visser D, Tolboom N, Smith CLC, Zwezerijnen G, van de Giessen E, et al. Validation of image-derived input function using a long axial field of view PET/CT scanner for two different tracers. EJNMMI Phys. 2024;11:25. 10.1186/s40658-024-00628-0. 38472680 10.1186/s40658-024-00628-0 PMC10933214

[24] O JH, Lodge MA, Wahl RL. Practical PERCIST: a simplified guide to PET response criteria in solid tumors 1.0. Radiology. 2016;280:576–84. 10.1148/radiol.2016142043. 26909647 10.1148/radiol.2016142043 PMC4976461

[25] Pitman EJG. A note on normal correlation*. Biometrika. 1939;31:9–12. 10.1093/biomet/31.1-2.9.

[26] Holm S. A simple sequentially rejective multiple test procedure. Scand J Stat. 1979;6:65–70.

[27] Levene H. Robust tests for equality of variances. In: Olkin I, editor. Contributions to probability and statistics: essays in honor of Harold hotelling. Palo Alto, CA: Stanford University Press; 1960. pp. 278–92.

[28] Meng H, Pan G, Peng Y, Yang J, Cui B, Zhang Y, et al. The reproducibility of [68Ga]Ga-FAPI-04 PET uptake parameters at 15 min and 60 min post-injection. Am J Nucl Med Mol Imaging. 2025;15:74–81. 10.62347/DCGC3250. 40401109 10.62347/DCGC3250 PMC12089092

[29] de Langen AJ, Vincent A, Velasquez LM, van Tinteren H, Boellaard R, Shankar LK, et al. Repeatability of 18F-FDG uptake measurements in tumors: a metaanalysis. J Nucl Med. 2012;53:701–8. 10.2967/jnumed.111.095299. 22496583 10.2967/jnumed.111.095299

[30] Lodge MA. Repeatability of SUV in oncologic (18)F-FDG PET. J Nucl Med. 2017;58:523–32. 10.2967/jnumed.116.186353. 28232605 10.2967/jnumed.116.186353 PMC5373499

[31] Ferrone CR, Marchegiani G, Hong TS, Ryan DP, Deshpande V, McDonnell EI, et al. Radiological and surgical implications of neoadjuvant treatment with FOLFIRINOX for locally advanced and borderline resectable pancreatic cancer. Ann Surg. 2015;261:12–7. 10.1097/SLA.0000000000000867. 25599322 10.1097/SLA.0000000000000867 PMC4349683

[32] Theijse RT, Stoop TF, Janssen QP, Prakash LR, Katz MHG, Doppenberg D, et al. Impact of a non-therapeutic laparotomy in patients with locally advanced pancreatic cancer treated with induction (m)FOLFIRINOX: trans-Atlantic Pancreatic Surgery (TAPS) Consortium study. Br J Surg. 2024. 10.1093/bjs/znae033. 38456678 10.1093/bjs/znae033 PMC10921832

[33] Ghidini M, Vuozzo M, Galassi B, Mapelli P, Ceccarossi V, Caccamo L, et al. The role of positron emission tomography/computed tomography (PET/CT) for staging and disease response assessment in localized and locally advanced pancreatic cancer. Cancers (Basel). 2021. 10.3390/cancers13164155. 34439307 10.3390/cancers13164155 PMC8394552

[34] Boellaard R. Repeatability of 18F-FDG uptake measurements in tumors: a metaanalysis. J Nucl Med. 2011;2:93S-100S. 10.2967/jnumed.110.085662. 10.2967/jnumed.111.095299 22496583

[35] Wahl RL, Jacene H, Kasamon Y, Lodge MA. From RECIST to PERCIST: evolving considerations for PET response criteria in solid tumors. J Nucl Med. 2009;50(Suppl 1):S122–50. 10.2967/jnumed.108.057307. 10.2967/jnumed.108.057307 PMC2755245 19403881

[36] Shankar LK, Huang E, Litiere S, Hoekstra OS, Schwartz L, Collette S, et al. <article-title update="added">Meta-analysis of the test–retest repeatability of [18F]-fluorodeoxyglucose standardized uptake values: implications for assessment of tumor response. Clin Cancer Res. 2023;29(1):143–53. 10.1158/1078-0432.CCR-21-3143. 36302172 10.1158/1078-0432.CCR-21-3143

