# Supplementary material for: Repeatability of semi-quantitative [68Ga]Ga-FAPI-46 PET/CT measurements in pancreatobiliary cancers: a test-retest study
Source: Eur J Nucl Med Mol Imaging. 2026 Jan 16;53(6):3827–37. doi: 10.1007/s00259-025-07738-6 (PMC13121203; doi:10.1007/s00259-025-07738-6)
Supplement: Supplementary file 1 — Supplementary Material 1 [file 259_2025_7738_MOESM1_ESM.docx]

**Supplemental materials**

**Repeatability of semi-quantitative [^68^Ga]Ga-FAPI-46 PET/CT measurements in pancreatobiliary cancers: a test-retest study**

**Authors:** Rutger B. Henrar ^1,2^, Matthijs C. F. Cysouw ^1,2^, Xavier Palard-Novello ^1,2,3^, Lothar A. Schwarte ^1,2^, Pieter G.H.M. Raijmakers ^1,2^, Lioe-Fee de Geus-Oei ^4,5,6^, Alexander L. Vahrmeijer ^4^, Geert Kazemier ^1,2^, Albert D. Windhorst ^1,2^, Ronald Boellaard ^1,2^, Maqsood Yaqub ^1,2^, Daniela E. Oprea-Lager^#1,2,7^, Rutger-Jan Swijnenburg^#1,2^

# authors contributed equally to this publication

**Affliliations:**

^1^ Amsterdam UMC location Vrije Universiteit Amsterdam, De Boelelaan 1117, Amsterdam, The Netherlands

^2^ Cancer Center Amsterdam, Imaging and Biomarkers, Amsterdam, The Netherlands

^3^ Univ Rennes, CLCC Eugène Marquis, INSERM, LTSI - UMR 1099, Rennes, France

^4^ Leiden University Medical Center, Albinusdreef 2, Leiden, the Netherlands

^5^ University of Twente, Drienerlolaan 5, Enschede, The Netherlands

^6^ Delft University of Technology, Mekelweg 6, Delft, The Netherlands

^7^ Radboud University Medical Center, Nijmegen, the Netherlands

**Corresponding author:**

Rutger-Jan Swijnenburg, MD, PhD

Department of Surgery, Amsterdam UMC location Vrije Universiteit Amsterdam

De Boelelaan 1117, 1081 HV, Amsterdam, The Netherlands

Tel: +31 20 444 4400

Email: r.j.swijnenburg@amsterdamumc.nl

**Supplemental Figure 1 A-C.**

Bland-Altman plots of the relative test-retest differences of the background tissues: blood pool (A), liver tissue (B) and muscle tissue (C). Each dot represents one patient (n=12). The patients are colour coded per tumour type: PDAC (red), perihilar CCA (blue) and intrahepatic CCA (green). One patient with a perihilar CCA was excluded from the analysis (open circle in figure B), due to an unsolved biliary obstruction which significantly impacted repeatability. The dashed line shows the mean difference (bias), while the shaded area indicates the 95% limits of agreement.


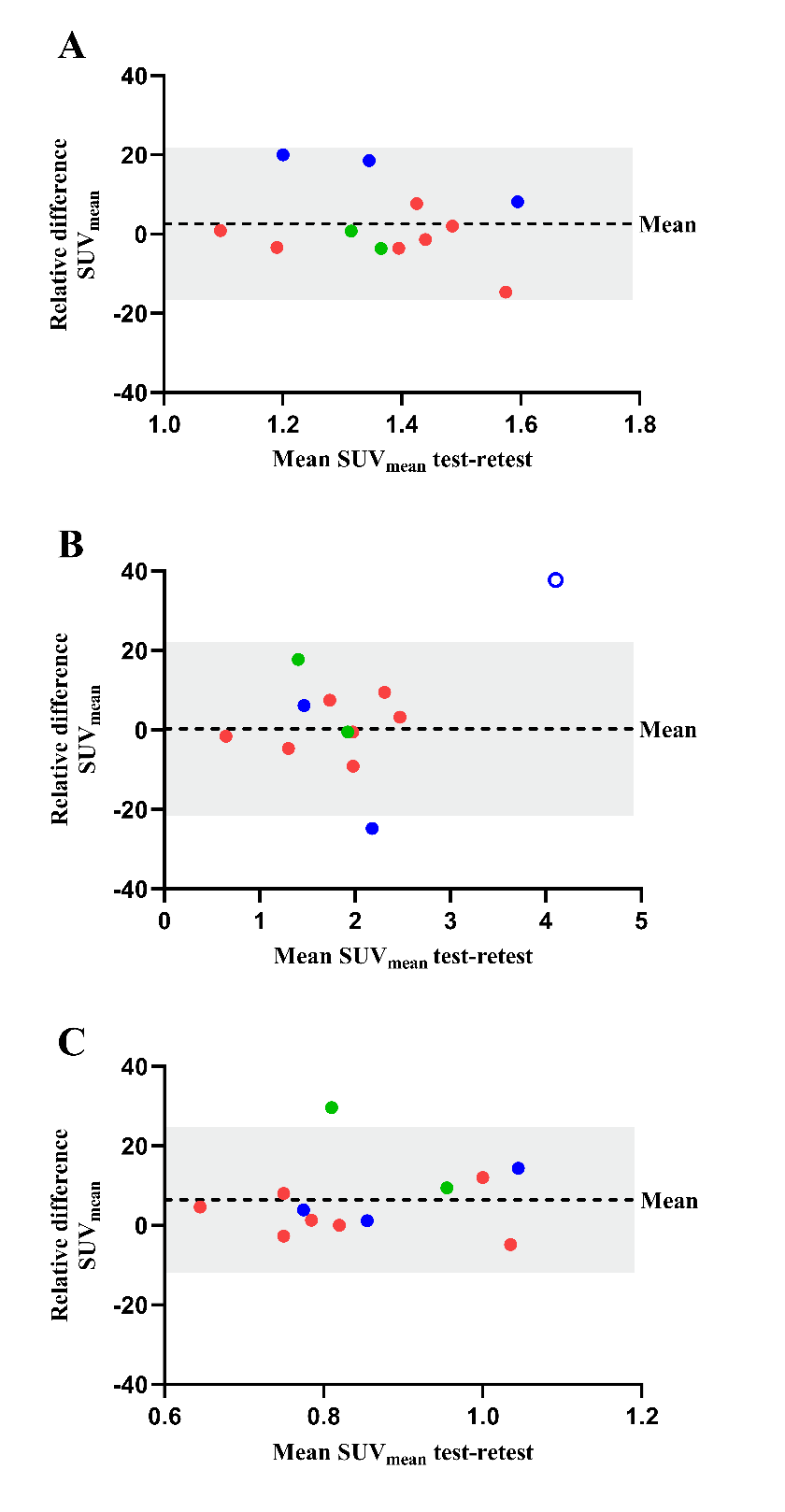


**Supplemental Figure 2 A-B.**

A representative example of a maximum intensity projection of a test (A) and retest (B) scan of a patient with resectable ductal adenocarcinoma of the pancreas, located in the tail.

**
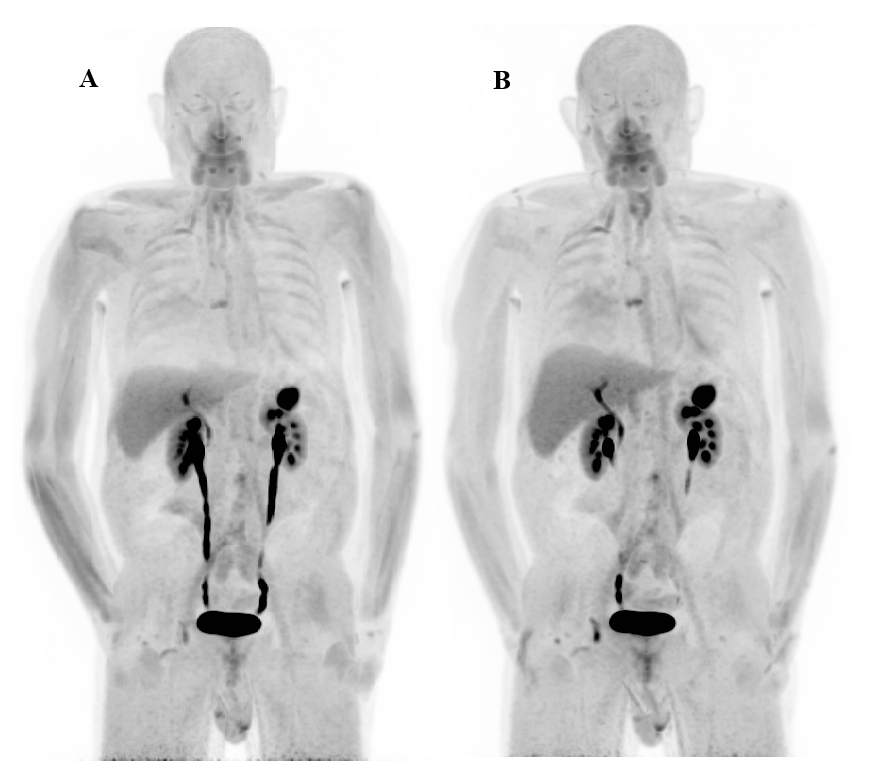
**

**Supplemental Figure 3 A-C.**

Bland-Altman plots of the relative test-retest differences of blood pool adjusted tumour-to-background ratios (TBR) shown against the mean tumour lesion volume: TBR_mean_ (A), TBR_peak_ (B) and TBR_max_ (C). Each dot represents one lesion (n=70). The X-axis is on a logarithmic scale. The dashed line shows the mean difference (bias), while the shaded area indicates the 95% limits of agreement.

**
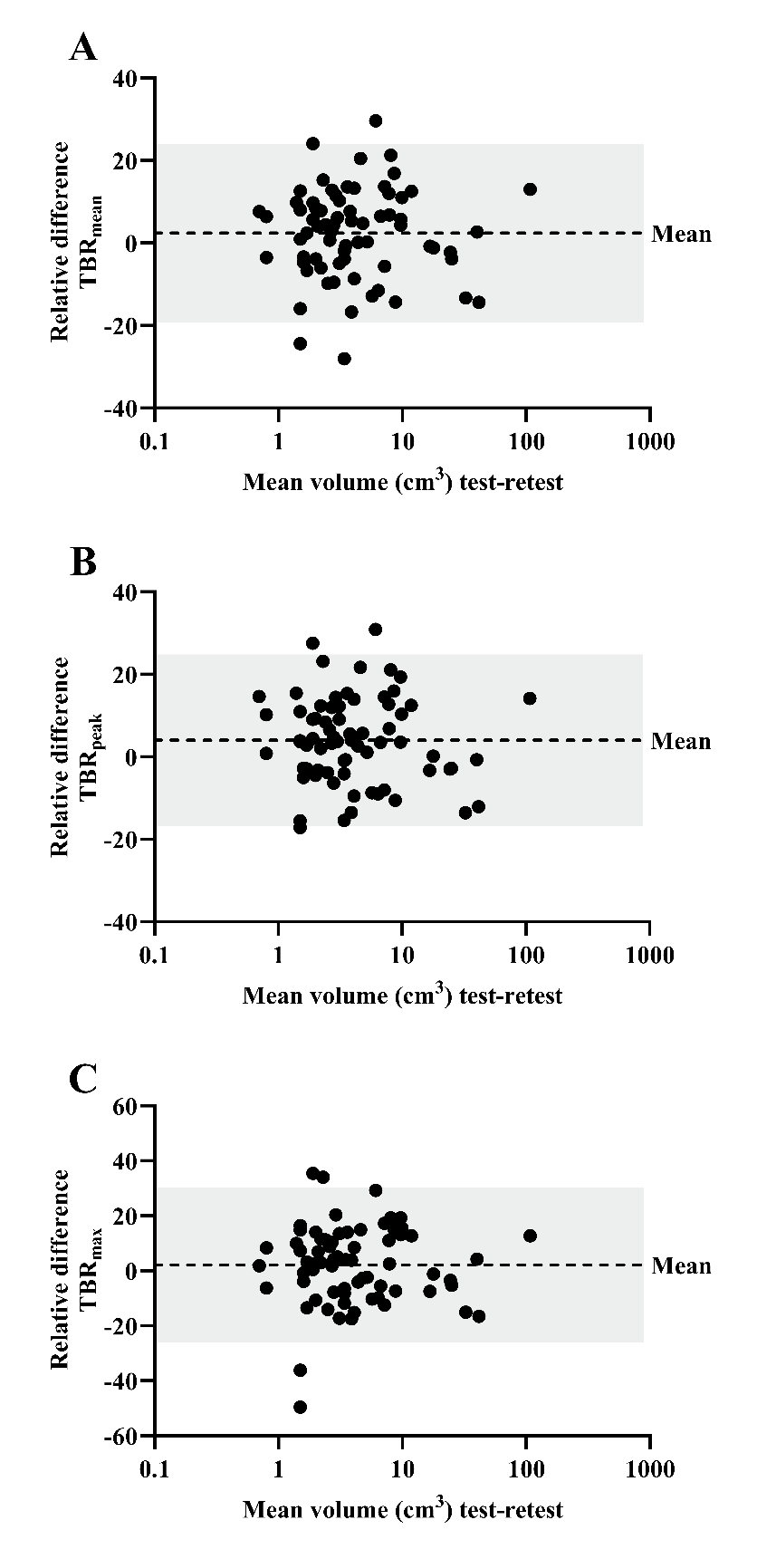
**

**Supplemental Figure 4 A-B.**

Bland-Altman plots of the relative test-retest differences of the total lesion uptake (TLU) metrics: SUV (A) and blood pool adjusted TBR (B). Each dot represents one lesion (n=70). The X-axis is on a logarithmic scale. The dashed line shows the mean difference (bias), while the shaded area indicates the 95% limits of agreement.

**
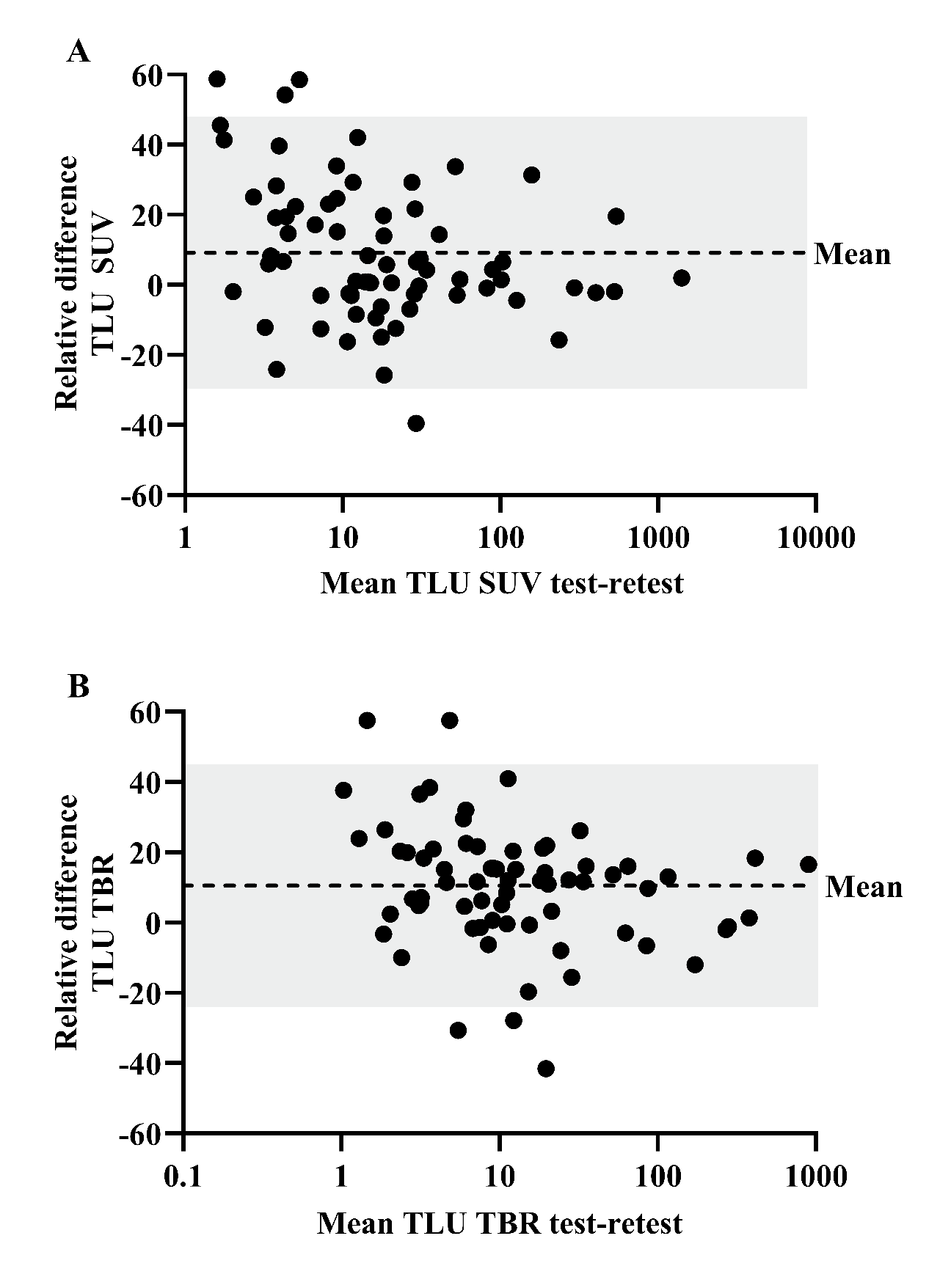
**

**Supplemental Figure 5 A-C.**

Bland-Altman plots of the relative test-retest differences of the patient based metrics: total tumour volume (TTV) (A), total tumour burden (TTB) of the SUV (B) and TTB of the blood pool adjusted TBR (C). Each dot represents one patient (n=12). The patients are colour coded per tumour type: red (PDAC), blue (perihilar CCA) and green (intrahepatic CCA). The X-axis is on a logarithmic scale. The dashed line shows the mean difference (bias), while the shaded area indicates the 95% limits of agreement.

**
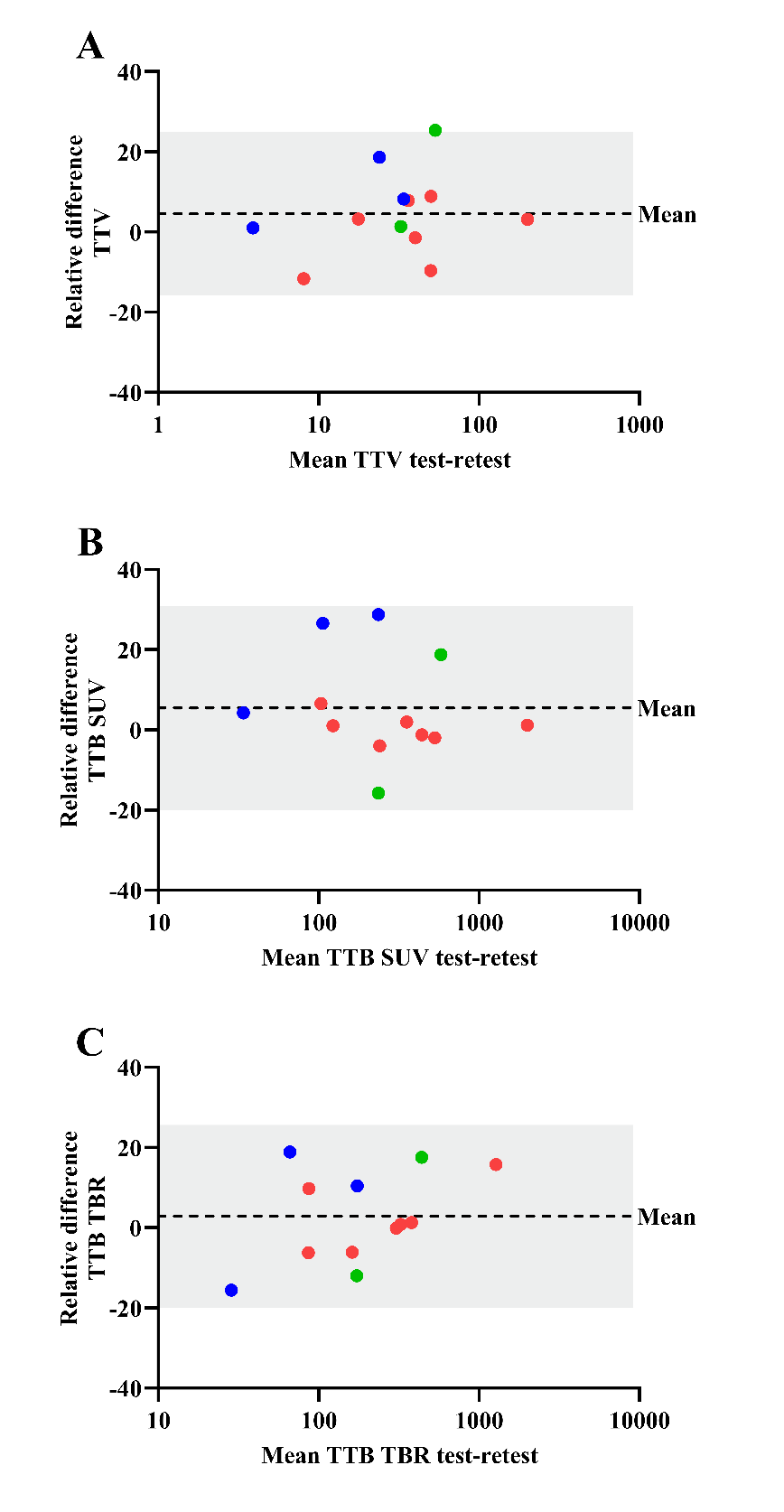
**

**Supplemental table 1.**

**Comparison of the relative test-retest differences of primary tumour lesions versus metastatic lesions.**

| **Parameter** | **Mean** | **SD** | **RC** | **Lower limit** | **Upper limit** | **ICC (95% CI)** |
| --- | --- | --- | --- | --- | --- | --- |
| **Primary tumours** |  |  |  |  |  |  |
| **Volume** | 5,7 | 12,0 | 23,5 | -17,8 | 29,2 | 0,99 (0,96 - 1,00) |
| **SUV_mean_** | 0,7 | 11,2 | 22,0 | -21,2 | 22,6 | 0,94 (0,82 - 0,98) |
| **SUV_peak_** | 2,2 | 12,5 | 24,5 | -22,3 | 26,8 | 0,95 (0,85 - 0,99) |
| **SUV_max_** | 1,1 | 12,6 | 24,7 | -23,5 | 25,8 | 0,95 (0,82 - 0,98) |
| **TBR_mean_** | -2,0 | 11,7 | 22,9 | -24,9 | 20,9 | 0,93 (0,78 - 0,98) |
| **TBR_peak_** | -0,4 | 12,3 | 24,1 | -24,5 | 23,7 | 0,95 (0,83 - 0,98) |
| **TBR_max_** | -1,5 | 13,0 | 25,5 | -26,9 | 23,9 | 0,94 (0,80 - 0,98) |
| **TLU SUV** | 6,4 | 14,7 | 28,8 | -22,4 | 35,2 | 1,00 (0,99 - 1,00) |
| **TLU TBR** | 3,7 | 12,9 | 25,3 | -21,5 | 29,0 | 0,98 (0,94 - 0,99) |
| **Metastases** |  |  |  |  |  |  |
| **Volume** | 8,2 | 21,2 | 41,6 | -33,4 | 49,8 | 0,94 (0,90 - 0,96) |
| **SUV_mean_** | 1,2 | 12,5 | 24,5 | -23,3 | 25,8 | 0,98 (0,96 - 0,99) |
| **SUV_peak_** | 2,9 | 12,4 | 24,3 | -21,5 | 27,3 | 0,98 (0,97 -0,99) |
| **SUV_max_** | 0,9 | 16,0 | 31,4 | -30,4 | 32,2 | 0,97 (0,95 - 0,98) |
| **TBR_mean_** | 3,6 | 10,8 | 21,2 | -17,7 | 24,8 | 0,97 (0,94 - 0,98) |
| **TBR_peak_** | 5,2 | 10,2 | 20,0 | -14,9 | 25,2 | 0,97 (0,93 - 0,99) |
| **TBR_max_** | 3,2 | 14,7 | 28,8 | -25,6 | 32,0 | 0,96 (0,93 - 0,98) |
| **TLU SUV** | 9,4 | 21,1 | 41,4 | -31,9 | 50,8 | 0,99 (0,98 - 0,99) |
| **TLU TBR** | 11,7 | 18,5 | 36,3 | -24,5 | 47,9 | 0,98 (0,95 - 0,99) |
| **Abbreviations:** SD: standard deviation, RC: repeatability coefficient, Lower and Upper limit: lower and upper limits of agreement, ICC: intraclass correlation coefficient (single measure, two way mixed model with absolute agreement), CI: confidence interval, SUV: standardised uptake value, TBR: tumour-to-background ratio (blood pool adjusted), TLU: total lesion uptake. | | | | | | |

**Supplemental table 2.**

**Comparison of the relative test-retest differences of confirmed versus non-confirmed malignant lesions.**

| **Parameter** | **Mean** | **SD** | **RC** | **Lower limit** | **Upper limit** | **ICC (95% CI)** |
| --- | --- | --- | --- | --- | --- | --- |
| **Confirmed lesions** |  |  |  |  |  |  |
| **Volume** | 5,4 | 12,9 | 25,3 | -19,8 | 30,7 | 0,99 (0,98 - 1,00) |
| **SUV_mean_** | 3,7 | 10,9 | 21,4 | -17,7 | 25,1 | 0,98 (0,95 - 0,99) |
| **SUV_peak_** | 5,1 | 11,7 | 22,9 | -17,9 | 28,0 | 0,98 (0,95 - 0,99) |
| **SUV_max_** | 3,7 | 13,5 | 26,5 | -22,8 | 30,1 | 0,98 (0,95 - 0,99) |
| **TBR_mean_** | 1,3 | 11,9 | 23,3 | -22,0 | 24,7 | 0,97 (0,93 -0,99) |
| **TBR_peak_** | 2,7 | 12,3 | 24,1 | -21,3 | 26,7 | 0,98 (0,95 - 0,99) |
| **TBR_max_** | 1,3 | 14,0 | 27,4 | -26,2 | 28,7 | 0,97 (0,93 - 0,99) |
| **TLU SUV** | 9,1 | 12,7 | 24,9 | -15,9 | 34,0 | 1,00 (0,99 - 1,00) |
| **TLU TBR** | 6,7 | 12,0 | 23,5 | -16,9 | 30,3 | 0,99 (0,96 - 0,99) |
| **Non-confirmed lesions** |  |  |  |  |  |  |
| **Volume** | 9,1 | 21,9 | 42,9 | -33,7 | 52,0 | 0,93 (0,89 - 0,96) |
| **SUV_mean_** | -0,1 | 12,4 | 24,3 | -24,4 | 24,3 | 0,98 (0,96 - 0,99) |
| **SUV_peak_** | 1,7 | 12,4 | 24,3 | -22,6 | 26,0 | 0,98 (0,97 - 0,99) |
| **SUV_max_** | -0,5 | 15,8 | 31,0 | -31,5 | 30,5 | 0,97 (0,94 - 0,98) |
| **TBR_mean_** | 2,9 | 10,7 | 21,0 | -18,1 | 23,9 | 0,96 (0,93 - 0,98) |
| **TBR_peak_** | 4,6 | 10,0 | 19,6 | -15,0 | 24,1 | 0,97 (0,93 - 0,99) |
| **TBR_max_** | 2,4 | 14,6 | 28,6 | -26,3 | 31,0 | 0,96 (0,92 - 0,98) |
| **TLU SUV** | 9,1 | 22,1 | 43,3 | -34,3 | 52,5 | 0,99 (0,98 - 1,00) |
| **TLU TBR** | 12,0 | 19,2 | 37,6 | -25,7 | 49,7 | 0,98 (0,95 - 0,99) |
| **Abbreviations:** SD: standard deviation, RC: repeatability coefficient, Lower and Upper limit: lower and upper limits of agreement, ICC: intraclass correlation coefficient (single measure, two way mixed model with absolute agreement), CI: confidence interval, SUV: standardised uptake value, TBR: tumour-to-background ratio (blood pool adjusted), TLU: total lesion uptake. | | | | | | |

**Supplemental table 3.**

**Comparison of the relative test-retest differences of metastatic lesions based on the mean volume**

**≤ 2.9 cm^3^ versus > 2.9 cm^3^.**

| **Parameter** | **Mean** | **SD** | **RC** | **Lower limit** | **Upper limit** | **ICC (95% CI)** |
| --- | --- | --- | --- | --- | --- | --- |
| **Lesions ≤ 2.9 cm^3^** |  |  |  |  |  |  |
| **Volume** | 12,6 | 21,1 | 41,4 | -28,8 | 54,0 | 0,77 (0,52 - 0,90) |
| **SUV_mean_** | 3,0 | 12,6 | 24,7 | -21,7 | 27,6 | 0,95 (0,91 - 0,98) |
| **SUV_peak_** | 5,1 | 12,9 | 25,3 | -20,1 | 30,3 | 0,97 (0,93 - 0,98) |
| **SUV_max_** | 3,6 | 18,3 | 35,9 | -32,2 | 39,5 | 0,94 (0,88 - 0,97) |
| **TBR_mean_** | 2,8 | 10,1 | 19,8 | -16,9 | 22,5 | 0,97 (0,93 - 0,98) |
| **TBR_peak_** | 4,8 | 10,0 | 19,6 | -14,9 | 24,5 | 0,98 (0,94 - 0,99) |
| **TBR_max_** | 3,3 | 17,0 | 33,3 | -30,0 | 36,6 | 0,95 (0,89 - 0,98) |
| **TLU SUV** | 15,5 | 20,8 | 40,8 | -25,2 | 56,2 | 0,95 (0,83 - 0,98) |
| **TLU TBR** | 15,3 | 17,3 | 33,9 | -18,7 | 49,3 | 0,93 (0,73 - 0,98) |
| **Lesions > 2.9 cm^3^** |  |  |  |  |  |  |
| **Volume** | 3,4 | 20,7 | 40,6 | -37,1 | 44,0 | 0,89 (0,76 - 0,95) |
| **SUV_mean_** | -0,6 | 12,4 | 24,3 | -25,0 | 23,7 | 0,98 (0,95 - 0,99) |
| **SUV_peak_** | 0,5 | 11,8 | 23,1 | -22,6 | 23,5 | 0,98 (0,95 - 0,99) |
| **SUV_max_** | -2,0 | 12,7 | 24,9 | -27,0 | 23,0 | 0,97 (0,94 - 0,99) |
| **TBR_mean_** | 4,4 | 11,8 | 23,1 | -18,6 | 27,4 | 0,96 (0,88 - 0,98) |
| **TBR_peak_** | 5,5 | 10,6 | 20,8 | -15,2 | 26,3 | 0,96 (0,87 - 0,98) |
| **TBR_max_** | 3,0 | 12,1 | 23,7 | -20,6 | 26,7 | 0,95 (0,90 - 0,98) |
| **TLU SUV** | 2,9 | 19,8 | 38,8 | -35,9 | 41,6 | 0,99 (0,97 - 0,99) |
| **TLU TBR** | 7,9 | 19,2 | 37,6 | -29,7 | 45,5 | 0,96 (0,91 - 0,98) |
| **Abbreviations:** SD: standard deviation, RC: repeatability coefficient, Lower and Upper limit: lower and upper limits of agreement, ICC: intraclass correlation coefficient (single measure, two way mixed model with absolute agreement), CI: confidence interval, SUV: standardised uptake value, TBR: tumour-to-background ratio (blood pool adjusted), TLU: total lesion uptake. | | | | | | |
